# A multidimensional framework for understanding problematic use of short video platforms: the role of individual, social-environmental, and platform factors

**DOI:** 10.3389/fpsyt.2024.1361497

**Published:** 2024-09-05

**Authors:** Sihan Xiong, Jing Chen, Nisha Yao

**Affiliations:** ^1^ School of Psychology, Shanghai University of Sport, Shanghai, China; ^2^ School of Kinesiology and Health, Capital University of Physical Education and Sports, Beijing, China

**Keywords:** short video platforms, problematic use, individual factors, social and environmental factors, platform factors

## Abstract

Short video platforms have rapidly become a prominent form of social media, but their problematic use is increasingly concerning. This review synthesizes existing research to propose a comprehensive framework that integrates individual, social-environmental, and platform-related factors contributing to this issue. Individual factors are categorized into distal (e.g., personality, psychopathology) and proximal (e.g., usage expectations, cognitive, emotional, and behavioral responses during use) categories, with distal factors often shaping proximal ones, which more directly influence usage behaviors. Social-environmental factors, such as family dynamics and peer interactions, along with platform-related features, also significantly impact the likelihood of problematic use. Beyond their direct effects, our framework emphasizes the importance of examining the combined effects of these factors, particularly through mediation and moderation processes. Mediation processes reveal how distal individual factors influence problematic use by shaping more immediate, proximal factors. Similarly, social-environmental influences and platform features may affect problematic use by modifying individual factors. Moderation processes further illustrate how individual characteristics or social-environmental factors may alter the strength of these relationships. Understanding these complex, multidimensional relationships is essential for developing effective interventions to mitigate the risks associated with problematic short video platforms use. Future research should explore these processes in greater depth.

## 1 Introduction

In recent years, short video platforms like TikTok have experienced rapid growth. As of February 2023, the monthly active user base of TikTok reached 1.8 billion, engaging approximately 18% of internet users aged 16 to 64 worldwide^1^. This surge has prompted concerns about potential negative impacts, leading researchers to focus on the phenomenon of problematic use of these platforms. Drawing from existing research on Internet addiction (1, 2), social media addiction (3, 4), and smartphone addiction (5), along with diagnostic criteria for gaming disorder, problematic use of short video platforms refers to persistent, uncontrollable usage of short video platforms that impairs daily functioning. This condition is characterized by preoccupation with using short video platforms (salience), increased usage to achieve the previous level of satisfaction (tolerance), negative emotions when usage is restricted (withdrawal), usage causing harm to important life areas (conflict), failed attempts to reduce usage (relapse), and usage to escape problems or improve mood [mood regulation; (2, 4, 6)].

The negative impacts of problematic use of short video platforms extend to both physical and mental health (7), including cognitive decline (8, 9), emotional issues (10), and functional impairment (7, 11, 12). Given these substantial health consequences, this review aims to identify its underlying risk factors. Understanding these factors and mechanisms that contribute to the problematic use of short video platforms has both theoretical and practical significance for preventing and addressing this issue, ultimately promoting healthy usage of short video platforms.

The contemporary model for problematic Internet use, the Interaction of Person-Affect-Cognition-Execution Model [I-PACE; (13, 14)], underscores the role of individual factors in its development and maintenance. This model also suggests that the core features of specific applications or sites (i.e., platform-related factors) play a role, as they must align with users’ motives. For instance, individuals seeking social interaction are more likely to use social networking sites. Similarly, other models, such as the cognitive-behavioral model of pathological Internet use (15) and the compensatory model (16), emphasize the significant role of individual factors in developing problematic use. The cognitive-behavioral model posits that maladaptive cognitions contribute to pathological Internet use, where individuals might develop irrational beliefs and engage in Internet use in response to these beliefs. On the other hand, the compensatory model suggests that individuals use the Internet to compensate for unmet offline needs, which can lead to excessive or problematic use. Furthermore, the Differential Susceptibility to Media Effects Model [DSMM; (17)] introduces various susceptibility factors affecting media use outcomes, including dispositional, developmental, and social susceptibilities. These factors influence individual media use and cognitive-emotional responses during use, explaining the differential impact of media on individuals. Taken together, existing models explaining (disordered) Internet or media use implicate individual, social-environmental, and platform-related factors, with an emphasis placed on individual factors.

Recent research suggests the need to differentiate between specific types of problematic online behaviors (18), with evidence showing that using short video platforms is the strongest predictor of social media addiction (19). Short video platforms, a novel form of social media, possess unique content modes, recommendation algorithms, and functional features designed to attract and retain users (20–27). These distinctive features are believed to facilitate problematic use (27), interacting with users’ personal characteristics to shape their usage behavior and outcomes (28). Consequently, existing models do not fully explain the phenomenon of problematic use of short video platforms, necessitating a specific framework for this issue. Montag et al. (28) emphasized the need to consider both individual and platform characteristics, yet their review primarily addressed general usage of short videos rather than the specific triggers of problematic use in this context. This review therefore aims to fill this gap by exploring the individual, social-environmental, and platform-related factors influencing problematic use behaviors on short video platforms. We seek to answer two key research questions: (1) What specific factors identified in empirical studies lead to problematic use of short video platforms? (2) How do these factors jointly contribute to escalated problematic use severity? Understanding these questions will provide clearer insights into the occurrence and prevention of problematic use of short video platforms.

Empirical studies have examined risk factors predisposing individuals to problematic use of short video platforms. Most research has focused on individual factors, with less attention on social-environmental and platform factors. Individual factors often include personality and traits, psychopathology, and social cognition, which may represent distal constructs less directly tied to problematic short video platforms use. More directly related individual factors include usage expectations and cognitive, emotional, and behavioral responses during platform use. Social-environmental factors, frequently studied in adolescents, usually focus on immediate contexts like family, school, and peers. Platform-related factors include both system and content characteristics that influence usage patterns. Research has also explored how individual, social-environmental, and platform factors jointly facilitate problematic short video platforms use. For instance, parental neglect increased the risk of problematic use more significantly in individuals with low self-control compared to those with high self-control, suggesting a moderating role of individual factors in the association between social-environmental factors and problematic short video platforms use (29). Additionally, system and content features of short video platforms can enhance the risk of problematic use by fostering a state of flow (23), illustrating the mediating role of individual factors between platform features and problematic use. Accordingly, we first reviewed individual (Section 2), social-environmental (Section 3), and platform factors (Section 4) affecting the problematic use of short video platforms. We then discussed the potential mechanisms by which these factors jointly influence problematic use (Section 5). Finally, we suggested future research directions and addressed the limitations of the current work (Section 6).

The current review makes several contributions to the existing literature. Firstly, it provides a comprehensive review of risk factors leading to problematic use of short video platforms. Secondly, it highlights the interplay between these factors, offering insights into how they collectively influence problematic short video platforms usage behavior. By forming a multidimensional framework that integrates individual, social-environmental, and platform factors, this review offers a better understanding of the development and maintenance of problematic short video platforms use. Finally, the review discusses the limitations of existing studies and suggests directions for future research to facilitate improving knowledge about this phenomenon.

## 2 Individual factors affecting problematic use of short video platforms

### 2.1 Personality and traits

Personality, as distal psychological constructs, may directly or indirectly influence problematic use of short video platforms through more proximal psychological structures. Research has shown a significant correlation between higher neuroticism and greater severity of problematic use of short video platforms (30). Furthermore, neuroticism was found to increase feelings of loneliness and boredom, which in turn increased problematic use (31). Given that individuals high in neuroticism often exhibit a tendency to experience negative emotions, the use of short video platforms may serve as a compensatory mechanism for emotion regulation (16). Additionally, lower agreeableness and conscientiousness have been linked to greater severity of problematic use of short video platforms (30). This suggests that individuals who struggle to maintain harmonious interpersonal relationships or lack self-discipline are more likely to overuse these platforms.

On the other hand, the relationship between extraversion and problematic use of short video platforms is less clear. Some research has found that individuals with higher levels of extraversion experience lower levels of loneliness, which further reduce their likelihood of problematic use (32). Relatedly, highly shy individuals were more likely to engage in excessive use, especially when they perceived high levels of stress or were motivated to compensate for unmet needs in real life (33). However, other studies suggested that extraverted individuals were more likely to develop problematic short video platforms use (6). It has been suggested that short video platforms provide a convenient means of self-presentation and promotion, which may motivate extraverted individuals to use these platforms more intensively (6). Supporting this view, higher levels of narcissism and need for admiration have been found to heighten problematic short video platforms use (34). These findings suggest that extraversion may influence the use of short video platforms through different pathways, leading to varied use outcomes.

Other trait factors, such as low trait mindfulness, were found to be significantly associated with problematic use of short video platforms (35), indicating the impact of lack of self-awareness on problematic use. Additionally, research indicated that individuals who adopted a fast life history strategy were more prone to developing an addiction to short video platforms, highlighting that those seeking immediate gratification and lacking long-term planning may be particularly vulnerable to this issue (36). Moreover, a lack of self-control has been recognized as a contributing factor to problematic short video platforms use (24, 29, 37, 38). When confronted with engaging short videos, individuals with poor self-control struggle to divert their attention, leading to increased use (24). Self-control may also serve to moderate the influence of other risk factors on problematic use (25, 29, 39). For instance, self-control was found to mitigate the impact of parental neglect on problematic use (29). Similarly, experiencing withdrawal symptoms (i.e., discomfort when use is interrupted) related to short video platforms use was more likely to result in problematic use among individuals with low self-control, such as those prone to procrastination (25). Thus, enhancing self-control is likely to reduce the probability of problematic short video platforms use directly or indirectly through alleviating the adverse effects of other risks.

In summary, personality and traits play a significant role in influencing problematic use of short video platforms. These studies explore the direct and indirect effects of personality and traits and their interaction with other factors, suggesting that they may exert influence through different pathways and contribute to varied outcomes. Future research is needed to clarify these relationships further.

### 2.2 Psychopathology

Psychopathology and its symptoms have been found to be significantly associated with problematic short video platforms use. Elevated levels of depression (7, 40), as well as high social anxiety (7, 11, 40) and low subjective well-being (7, 41), were predictive of more severe problematic use of short video platforms. Depression and social anxiety may interfere with individuals’ normal emotional processing and heighten their distress intolerance. As a result, these individuals were more likely to turn to short video platforms for emotional regulation, leading to excessive use (40). Individuals with lower levels of subjective well-being were found to have a heightened fear of missing out (FoMO)—a concern about missing out on rewarding experiences or failing to keep up with others. This, in turn, contributed to their excessive use of short video platforms (41). Hence, mental health conditions, such as depression, social anxiety, and low subjective well-being may negatively affect emotional and cognitive processes, potentially triggering excessive use of short video platforms as a means of self-regulation (42).

### 2.3 Social cognition

Given the social attributes of short video platforms, social cognition and related factors may influence problematic use of short video platforms. Feelings of loneliness (6, 7), social isolation (26), and a lack of offline social support combined with a reliance on seeking online social support (43) were all found to contribute to the problematic use of short video platforms. Individuals lacking offline social support often exhibited higher levels of emotional suppression and relational needs, making them more likely to use short video platforms for emotional regulation and to fulfill their social needs (43). Moreover, those experiencing social isolation tended to develop stronger attachments to the user community on short video platforms (26), fostering connectedness that may lead to problematic use.

Additionally, short video platforms, characterized by their user-generated content (UGC), facilitate social comparison, an aspect that has a significant impact on problematic use. Social comparison can take place in terms of abilities, such as personal achievements and qualifications, or opinions, such as views and attitudes, which helps individuals to evaluate and understand themselves. Individuals can also engage in upward social comparison, comparing themselves to those perceived as better, or downward social comparison, comparing themselves to those perceived as worse (44). Research showed that ability-based social comparison and upward social comparison were significantly correlated with problematic use of short video platforms (44). This suggests that individuals who frequently engage in these types of social comparisons are more likely to immerse themselves in the vast amount of UGC available on these platforms, leading to problematic use.

### 2.4 Usage expectation

The Uses and Gratifications Theory (45) suggests that individuals develop motivations to use social media based on their needs and actively choose specific social media platforms to satisfy those needs. Short video platforms in particular can satisfy a wide range of user needs, thereby stimulating different use expectations (46–49). For example, they offer opportunities for information seeking and entertainment, allowing users to stay informed and amused. They also cater to trend-following and novelty-seeking behaviors, as users are drawn to new and popular content. Additionally, these platforms can serve as a means of stress relief and reality escape, providing a temporary break from everyday pressures. Users may also turn to short video platforms for self-presentation and expression, showcasing aspects of their identity and creativity. Moreover, these platforms can fulfill needs related to self-compensation, where users seek to make up for perceived deficiencies in their lives. Social identity and social interaction are also important motivators, as users engage with content and connect with others. Research showed that certain use expectations, such as reality escape, self-compensation, and social interaction, were linked with problematic use of short video platforms (33, 39, 48, 50). Specific expectations like escapism and social interaction have been found to enhance the flow experience and sense of belonging during use, further increasing the likelihood of problematic use (50). In addition, perceived stress may heighten the motive for self-compensation (33) and escapism (48), subsequently leading to problematic use. These findings highlight the potential detrimental effects of compensatory use of short video platforms to avoid real-life challenges, compensate for perceived deficiencies, or fulfill their social needs.

### 2.5 Cognitive, emotional, and behavioral responses

Individuals exhibit diverse cognitive, emotional, and behavioral responses when confronted with external environments and internal experiences, influencing their engagement with short video platforms. Research has demonstrated that perceived stress contributes to an increase in problematic use of short video platforms (33, 48, 51). Specifically, perceived stress has been linked to extended usage durations on short video platforms (51) and self-compensatory motives (33), consequently elevating the likelihood of problematic use. Perceived stress was also found to activate negative beliefs about worry (e.g., the belief that worry is uncontrollable), which in turn led individuals to use short videos as a form of escapism, ultimately leading to problematic use behaviors (48). In addition to perceived stress, factors such as boredom (52), distress intolerance (40), emotion dysregulation (53), and FoMO (41, 54) also render individuals more susceptible to problematic use of short video platforms.

Other studies have investigated the impact of cognitive, emotional, and behavioral responses during short video platforms use. A consistent finding across studies was that a greater experience of flow while using short video platforms was associated with an increased risk of problematic use (12, 51, 52, 55–57). The flow experience, reflecting a deeply engaged state of activity, directly contributed to problematic short video platforms use (12, 51, 52, 55, 58, 59). Researchers have examined various dimensions of the flow experience, such as enjoyment, concentration, time distortion, curiosity, and telepresence, and their impact on problematic use of short video platforms. These dimensions involve experiencing pleasure, immersion while using, losing a sense of time, wanting to follow up on platform content, and a sense of being in the virtual world created by the platforms, respectively. For instance, one study found that concentration and time distortion were associated with increased problematic use (56). Another study revealed that telepresence had a stronger effect on problematic use relative to the overall flow experience (57). Additionally, several antecedent factors contribute to the flow experience when using short video platforms, including specific characteristics of short video platforms [e.g., low interaction costs; (20, 23)] and escapist motivations (50).

Beyond flow experiences, higher satisfaction with short video platforms (21), stronger feelings of pleasure and withdrawal associated with use (25), and greater attachment to the platform and its user community (26, 50) have also been found to increase the likelihood of developing problematic use. Furthermore, the way individuals use short video platforms influences the development of problematic use. For instance, individuals with higher usage intensity (6, 11, 24, 51) and those who passively watch short videos (40) are more likely to develop problematic use.

Use expectations and cognitive, emotional, and behavioral responses may represent factors proximal to the use of short video platforms. To further our understanding, future research could explore their antecedents (20, 23, 33, 51), contributing to a more comprehensive understanding of problematic use of short video platforms.

### 2.6 Discussion

There has been a growing body of research focusing on how individual factors affect the development of problematic short video platforms use. These factors can be categorized into distal factors, such as personality and traits, psychopathology, and social cognition, and proximal factors, such as use expectations and cognitive, emotional, and behavioral responses. Distal factors may shape more immediate, proximal factors and their influence on short video platforms use, leading to varied outcomes. In alignment with this view, existing literature has not only examined the direct effects of individual factors but also explored how personality, psychopathology, and social cognitive processes contribute to problematic use both directly and indirectly through their influence on use expectations and cognitive, emotional, and behavioral responses (e.g., 34, 40, 41, 43). Furthermore, research suggests a nuanced relationship between proximal individual factors. For instance, some studies have investigated how use expectations influence responses during platform use, such as how escapism increases the flow experience during use, leading to problematic use (50). On the other hand, other studies have examined how such responses in daily life, like perceived stress, affect use expectations and contribute to problematic use (33). Additionally, research has shown that distal risks, such as self-control, can moderate the influence of proximal risks on problematic use (25, 39). We believe that integrating these insights into a hierarchical model provides a robust structure for future research, informing pathways for potential interventions to mitigate the negative impacts of excessive short video platforms use.

## 3 Social-environmental factors affecting problematic use of short video platforms

### 3.1 Family environment

Existing studies have explored various aspects of how the family environment influences the problematic use of short video platforms among youth. One area of focus is parental phubbing, where parents’ preoccupation with their phones has been linked to increased problematic use of short video platforms by adolescents (35, 60). When parents prioritized their phones, the adolescents may experience feelings of relative deprivation, perceiving themselves as less important than the devices. This negative experience could further increase their risk of problematic use of short video platforms (60). However, some studies have not found a significant impact of parental phubbing on adolescent problematic use of short video platforms after accounting for factors such as social anxiety and academic pressure (11), indicating that this relationship requires further investigation.

Beyond phubbing, studies have also investigated the effects of parental neglect, various parenting styles, and the specific dynamics of parent-child attachment and monitoring. Evidence indicated that more severe parental neglect correlated with an increased likelihood of problematic use of short video platforms; further analysis suggested that parental neglect may increase adolescents’ alexithymia, making them more prone to problematic use (29, 61). It has also been demonstrated that adolescents with problematic use of short video platforms often experienced parenting styles characterized by rejection, overprotection, and lack of warmth (7). Research showed that harsh parenting may exacerbate adolescents’ difficulties in emotion regulation, contributing to problematic use of these platforms (53). Additionally, adolescents’ attachment anxiety towards their parents, as well as poor parent-child relationship, was positively correlated with their addiction to short video platforms (35, 54). It has been revealed that adolescents with high attachment anxiety often have unmet social needs, leading to greater FoMO, characterized by an intense apprehension that others are experiencing rewarding activities without them. This, in turn, drives them to constantly engage with short video platforms to stay connected and avoid missing out on social experiences (54). On the other hand, parental monitoring has been shown to reduce adolescents’ problematic use of short video platforms (35, 56). Active parental mediation—parents discussing media use with their children—can moderate the influence of flow experience during platform use, preventing it from escalating into addiction (56). This suggests that effective parental involvement can prevent adolescents from progressing into addiction to short video platforms.

These findings demonstrate the complex interplay between family dynamics and youth problematic use of short video platforms. They highlight the need for a nuanced understanding of how different facets of the family environment contribute to problematic use. Furthermore, these findings suggest that interventions aimed at reducing problematic use should address both the behavioral and emotional aspects of parent-child interactions. For example, strategies that promote healthy technology use among parents and effective monitoring, coupled with support for improving family relationships, may help mitigate excessive use of short video platforms.

### 3.2 School environment

Research showed that school connectedness, a close emotional connection with schools, peers, and teachers, could reduce the risk of problematic use of short video platforms among adolescents (35). Furthermore, higher levels of school connectedness may attenuate the negative effect of parental neglect on adolescents’ problematic use of short video platforms (29). Conversely, academic burnout—characterized by a feeling of exhaustion from study demands, leading to disengagement from studies and a sense of incompetence as a student—was associated with an increased likelihood of problematic use of short video platforms among adolescents (7, 11). It is likely that high school connectedness provides social support and a sense of belonging, enhancing self-regulation and reducing the need for external distractions. In contrast, school burnout increases stress and lowers self-efficacy, leading adolescents to use short video platforms as a coping mechanism for academic pressures. These findings suggest that enhancing students’ emotional bonds with their school and addressing academic burnout could serve to reduce students’ risk of problematic use.

### 3.3 Peer factors

Peer influence plays a significant role in adolescents’ use of short video platforms. Research found that adolescents who felt peer pressure to use mobile phones were more likely to engage in problematic use of short video platforms (35). The pressure to affiliate with peers may drive individuals to use mobile phones more frequently, leading to problematic use of short video platforms through their phones. Additionally, experiences of being bullied or affiliating with deviant peers increased the likelihood of problematic use of short video platforms among adolescents (7, 35). However, positive peer communication can buffer against the negative effects of adverse family factors (e.g., parental phubbing) on the problematic use (60). Given these findings, peer influence, both positive and negative, significantly impacts adolescents’ use of short video platforms. Interventions aimed at reducing peer pressure and fostering supportive peer communication may help to mitigate the risk of problematic use among adolescents.

### 3.4 General social environment

The broader social environment also impacts adolescents’ use of short video platforms. Higher cumulative social-environmental risk— encompassing factors like poor parent-child relationships, poor parental relationships, low parental monitoring, parental phubbing, low school connectedness, poor teacher-student and peer relationships, peer pressure to use mobile phones, being bullied, and affiliation with deviant peers—was associated with more severe problematic use of short video platforms (35). However, research on the influences of macro social-environmental factors on the problematic use of short video platforms is still limited and awaits investigation.

### 3.5 Discussion

Current research on the social-environmental factors influencing problematic short video platforms use has mainly focused on family, school, and peer influences, with an emphasis on adolescents. Findings support the significant impact of these factors and suggest the potential for interventions that enhance family dynamics, school connectedness, and peer support to reduce the risk of excessive use of short video platforms. Furthermore, some research has shown that socio-environmental factors interact with individual factors (e.g. the interaction between flow experience and parental monitoring; 56) or influence problematic use behaviors by influencing internal experiences (e.g., harsh parenting influenced use by increasing emotion dysregulation; 53). Additionally, social-environmental factors in one domain could moderate the influence of social-environmental factors in other domains, such as school connectedness as well as peer communication may buffer against the negative impact of adverse family environment on problematic use (29, 60). These findings suggest the need to integrate these different factors into a comprehensive model to understand the pathways leading to problematic use.

## 4 Platform factors affecting problematic use of short video platforms

Researchers have analyzed several key features of short video platforms, such as TikTok, and proposed mechanisms through which these features lead to problematic use (27). Specifically, short video platforms adopt a UGC model where any life detail can become platform content, thereby covering a wide range of users’ interests. These platforms combine algorithms with a deep understanding of user preferences to efficiently deliver personalized content and continuously optimize content screening and pushing mechanisms. This algorithm-driven approach allows users to discover content beyond their typical interests, broadening their self-perception. Further, the platform design facilitates low-cost interaction, enabling algorithms to help users select content and continuously push it, while users only need to make judgments on the selected content. These features promote users’ engagement with short video platforms. The more users engage, the more accurate the algorithms become, which, in turn, enables more precise content pushing and exacerbates the risk of problematic use. Thus, there may be a closed-loop relationship between users’ problematic use and algorithm optimization (27).

Accumulating empirical studies have focused on how the characteristics associated with short video platforms affect users’ problematic usage behavior. These characteristics are categorized into system and content-related factors, with processes linking them to problematic use being discussed below.

### 4.1 System factors

System factors refer to the characteristics of the short video platforms, including personalized content delivery, ease of use, instant gratification of user needs, social features, and multimodality. These system characteristics may contribute to problematic use (20, 21, 23, 26). Among these factors, personalized content delivery has been most extensively studied (20, 21, 26, 37). Studies indicated that personalized content delivery not only directly increased the risk of problematic use (21, 37) but also amplified it by enhancing users’ attachment to the platform (26). Meanwhile, multimodality (e.g., diverse special effects afforded by short video platforms) and low-cost interactions between users and the system (e.g., ease of use, instant gratifications) can enhance users’ flow experience while using the platform, thereby increasing the risk of problematic use (20, 23). Finally, the social features of the platform (e.g., friending, liking, status updates, comments, and comparing to others) may induce positive feelings during use and negative feelings during interrupted use, which may then contribute to excessive use (25).

### 4.2 Content factors

Content factors refer to the characteristics of the content on short video platforms, such as short duration, accessibility, informativeness, and entertainment. These content characteristics may also promote problematic use (23). Studies have indicated that the brief duration of content, which demands less attention while satisfying needs in a shorter timeframe, along with the accessibility of diverse and freely available content, and the entertaining nature of short videos, could escalate the risk of problematic use of short video platforms (22). Furthermore, the entertaining nature of the content can heighten users’ attachment to the platform, thereby increasing problematic use severity (26). Finally, the informativeness and entertainment provided by short videos can enhance user satisfaction, potentially leading to excessive use (21).

### 4.3 Discussion

Given these unique features of short video platforms, more research is needed to incorporate platform factors into studies on their problematic use. Notably, although platform features play a role in increasing user engagement and may contribute to problematic use, not all users experience problematic use. This suggests that when considering the factors affecting the problematic use of short video platforms, researchers must also consider individual characteristics and the environmental context in which users live. For example, existing literature suggests that platform factors could increase the risk of problematic use by affecting cognitive, emotional, and behavioral responses during use (20, 23, 25, 26). Therefore, a multidimensional model integrating the effect of individual, social-environmental, and platform factors is proposed to explain problematic usage behavior.

## 5 Potential mechanisms of problematic use of short video platforms

### 5.1 Existing theoretical models

Existing theoretical models provide a foundation for understanding Internet addiction, which can inform our analysis of problematic use of short video platforms. The I-PACE model (13, 14) suggests that an individual’s personal characteristics (Person) influence their cognitive and emotional responses (Affect and Cognition) when faced with specific external and internal stimuli, affecting their usage behavior towards particular online applications. Additionally, lower executive functions (Execution) may exacerbate the impact of these personal characteristics, cognitive, and emotional factors on usage behavior. The I-PACE model thus provides a comprehensive framework for understanding the nuanced interplay between various individual factors. Similarly, the cognitive-behavioral model of pathological Internet use (15) proposes a pathway leading from distal risk factors (e.g., psychopathology) to proximal risk factors (e.g., maladaptive cognitions such as rumination), which then leads to pathological use. In addition, Kardefelt-Winther (16) suggested that using the Internet as a means of compensating for negative experiences in daily life could result in problematic use. These models indicate that within the domains of individual factors, various pathways and interactions can lead to problematic Internet use behaviors.

Beyond individual risk factors, the DSMM considers a broader range of susceptibility factors affecting media use outcomes (17). The DSMM suggests that dispositional (e.g., personality, attitudes, and motivation), developmental (e.g., cognitive, emotional, and social developmental stages), and social factors (e.g., influences from family, peers, schools, and (sub)cultures) influence individual media use behaviors. These factors further shape within-person experiences resulting from media use. Although this model is not specifically designed to understand problematic media use, it underscores the necessity of integrating factors across various domains to comprehend media use comprehensively.

While these models provide valuable insights, they primarily focus on general Internet or media use. Montag et al. (28) reviewed factors influencing the use of short video platforms and highlighted the need to consider both individual factors and platform characteristics when studying short video platforms use. However, their study focused on general usage behaviors without thoroughly investigating the factors causing problematic use. Thus, building on existing theoretical frameworks and a thorough review of the literature on problematic short video platforms use, we propose a multidimensional framework for understanding this phenomenon.

### 5.2 Proposed framework

**Figure 1:**
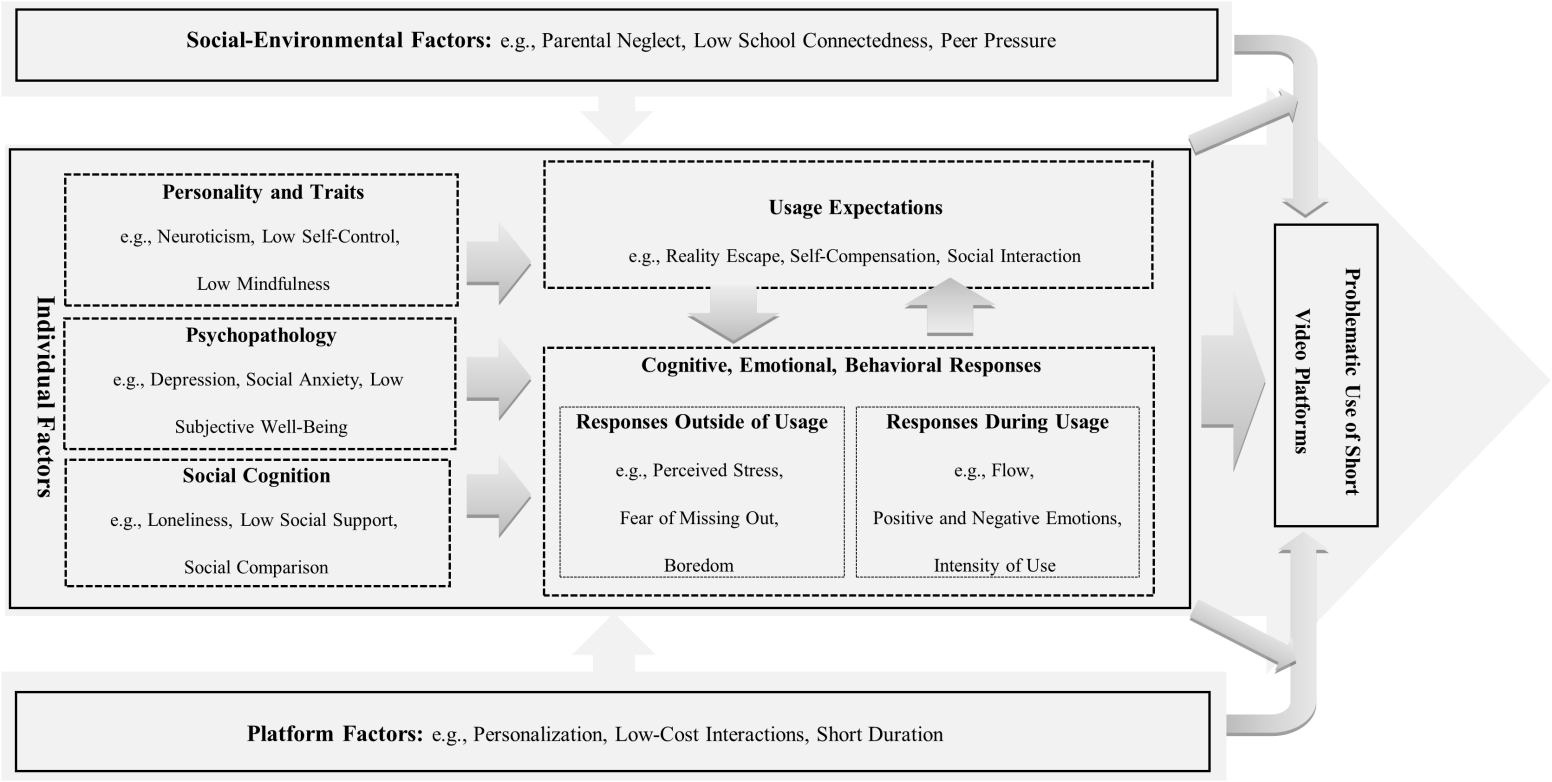
Pathways through which individual, social-environmental, and platform factors influence problematic use of short video platforms.

This review synthesizes the pathways through which individual, social-environmental, and platform factors influence the problematic use of short video platforms (see **Figure 1**). **Table 1** lists factors influencing the problematic use of short videos platforms. Existing research on individual factors is more extensive compared to research on social-environmental and platform-relate factors. Within the domain of individual factors, we distinguish between distal individual factors, such as personality and traits, psychopathology, and social cognition, and relatively proximal individual risk factors, such as usage expectations and cognitive, emotional, and behavioral responses. Distal individual factors may exert a broader influence, as evidenced by their impact on various behavioral addiction problems (13, 14). Proximal individual factors, on the other hand, may be more closely related to the use of short video platforms. Regarding social-environmental factors, existing studies have examined the influence of family, peers, and schools, along with their cumulative influence on the risk of problematic short video platforms use. For platform-related factors, the influence of features related to the platform and the content on the platform were examined.

**Table 1:** Risk factors of Problematic Use of Short Video Platforms.

Category	Variables	References
Individual Factors		
Personality and Traits	Extraversion; Shyness	Smith and Short (6); Mao and Jiang (32); Liu et al. (33)
	Neuroticism	Li et al. (30); Mao and Jiang (31)
	Agreeableness; Conscientiousness	Li et al. (30)
	Mindfulness	Liu et al. (35)
	Self-control; Inhibitory control; Procrastination	Su et al. (24); Tian et al. (25); Liu et al. (29); He et al. (38); Zhang et al. (39)
	Life history strategy	Wang et al. (36)
Psychopathology	Depression	Chao et al. (7); Yao et al. (40)
	Social anxiety	Chao et al. (7); Mu et al. (11); Yao et al. (40)
	Low subjective well-being	Chao et al. (7); Chung (41)
Social Cognition	Loneliness	Smith and Short (6); Chao et al. (7); Mao and Jiang (31)
	Social isolation	Zhang et al. (26)
	Social support	Yang et al. (43)
	Social comparison	Lewin et al. (44)
Usage Expectation	Self-compensation	Liu et al. (33); Zhang et al. (39)
	Social interaction	Miranda et al. (50)
	Escapism	Sun et al. (48); Miranda et al. (50)
Cognitive, Emotional, and Behavioral Responses	Perceived stress	Chao et al. (7); Liu et al. (33); Sun et al. (48); Huang et al. (51)
	Boredom	Mao and Jiang (31); Yao et al. (40); Lu et al. (52)
	Emotion dysregulation; Distress intolerance	Yao et al. (40); Wang et al. (53)
	Fear of missing out	Chung (41); Wang et al. (54)
	Flow	Han et al. (58); Ye et al. (12); Huang et al. (20); Qin et al. (23); Miranda et al. (50); Huang et al. (51); Lu et al. (52); Nong et al. (55); Qin et al. (56); Roberts and David (57); Zhou and Lee (59)
	User satisfaction; Feeling of withdrawal/enjoyment	Jingga et al. (21), Tian et al. (25)
	A sense of belonging/attachment to the user community and the platform	Zhang et al. (26), Miranda et al. (50)
	Usage behavior	Smith and Short (6); Mu et al. (11); Su et al. (24); Liu et al. (35); Yao et al. (40); Huang et al. (51)
Social-Environmental Factors		
Family Environment	Parental phubbing	Mu et al. (11); Liu et al. (35); Wang and Lei (60)
	Parental neglect	Liu et al. (29); Li et al. (61)
	Parenting styles and parent-child attachment/relationship	Chao et al. (7); Liu et al. (35); Wang et al. (53); Wang et al. (54)
	Parental monitoring	Liu et al. (35); Qin et al. (56)
School Environment	School connectedness	Liu et al. (29); Liu et al. (35)
	Academic burnout/stress	Chao et al. (7); Mu et al. (11)
Peer Factors	Negative peer influence (peer pressure, bullying, association with deviant peers)	Chao et al. (7); Liu et al. (35)
	Positive peer influence (peer communication)	Wang and Lei (60)
General Social Environment	Cumulative social-environmental risks	Liu, Yang et al. (35)
Platform-related Factors		
System Factors	Personalized content delivery; Ease of use; Instant gratification of user needs; Social features; Multimodality	Huang et al. (20); Jingga et al. (21); Qin et al. (23); Tian et al. (25); Zhang et al. (26)
Content Factors	Short duration; Accessibility; Informativeness; Entertainment	Jingga et al. (21); Karunakaran et al. (22); Qin et al. (23); Zhang et al. (26)

These factors could all directly influence the risk of problematic short video platforms use. However, to better understand the processes leading to problematic use, the joint effects of these factors should also be considered. These joint effects can be delineated using mediation and moderation effects. Regarding mediation effects, distal individual factors may influence problematic use by shaping proximal individual factors. This pathway has been reflected in existing research, such as how depression and anxiety contribute to problematic short video platforms use through elevated distress intolerance (40). Additionally, a lack of offline social support can give rise to a greater need for relatedness, which increases problematic short videos use severity (43). Beyond individual risks, both social-environmental and platform factors likely influence use behaviors and outcomes by shaping internal experiences (i.e., individual factors). For example, existing research has found that harsh parenting could disrupt adolescents’ emotion regulation, leading to their problematic use of short videos potentially as a means to compensate for dysregulated emotions (53). Additionally, the system features such as low-cost interactions offered by short video platforms can enhance users’ flow experience while using, thereby increasing the risk of problematic use (23). These mediation pathways suggest the existence of multiple, hierarchical processes leading to problematic short video platforms use.

Regarding moderation effects, individual factors may moderate the relationships between other individual, social-environmental, and platform factors and the problematic use of short video platforms. The moderating role of individual factors may reflect individual differences in responding to specific internal experiences, social environments, as well as platform features. For example, highly shy individuals are more likely to develop problematic short video platforms use when experiencing stress (33). In addition, it was found that individuals with high self-control could ameliorate the negative effects of parental neglect on increasing the risk of problematic use compared to those with low self-control (29). Besides, social-environmental factors may also play a moderating role, demonstrating how social environments can buffer against or worsen the negative effects of other risk factors. For instance, effective parental monitoring can mitigate the progression from flow experience during use to problematic use (56), while peer communication can reduce the adverse effect of parental phubbing on problematic use (60).

In sum, our proposed framework integrates individual, social-environmental, and platform factors into a comprehensive model to better understand the problematic use of short video platforms. It emphasizes recognizing how each factor—whether individual, social-environmental, or platform-related—could directly influence the risk of developing problematic use. More importantly, understanding the mediating pathways where distal individual factors such as personality shape proximal factors like usage expectations and emotional responses, and how social-environmental and platform-related influences affect within-person responses, is crucial. Additionally, considering the moderating influences where risk factors in one domain alter the impact of other risk factors on usage behavior helps elucidate how they collectively contribute to the development of problematic use. This multidimensional approach offers a comprehensive understanding of the complex dynamics at play. By identifying and addressing these factors, we can develop more effective interventions and preventive measures to mitigate the risks associated with problematic short video platforms use.

## 6 Conclusion and outlook

This review integrates the individual, social-environmental, and platform factors affecting problematic use of short video platforms and discusses the pathways through which these factors influence such use (**Figure 1**). Future research should further analyze how distal individual factors, such as personality and traits, influence problematic use through proximal individual factors, such as usage expectations and in-use responses. Additionally, it is important to explore how social-environmental and platform factors impact problematic use by influencing these individual factors, forming a multi-factor hierarchical model. Moreover, investigating the moderating role of risk factors across these domains is essential. Understanding how individual, social-environmental, and platform-related characteristics alter the impact of other risk factors on the transition from regular use to problematic use can provide valuable insights. This would enhance our comprehension of the conditions that promote or mitigate the progression to problematic use, thereby informing more effective interventions and preventive measures.

However, there are specific limitations in the proposed framework. Most existing empirical studies are based on cross-sectional surveys and cannot explain causal relationships. Thus, this framework lacks the ability to determine the direction of influence between risk factors and problematic use. Besides, existing research on risk factors of problematic use of short video platforms is relatively limited, suggesting that the derived framework could be modified and extended with growing studies in this area. Furthermore, most current studies focus on adolescents and young adults as participants, which does not cover all age groups. Therefore, it is uncertain whether this model has the same generalizability across all user groups. Finally, this framework illustrates the impact of individual, social-environmental, and platform factors on problematic use of short video platforms but does not reflect the reverse effect of the latter on the former. Problematic use of short video platforms can have a negative impact on an individual’s physical and mental health (8, 62, 63), potentially exacerbating the influence of the individual, environmental, and platform factors, creating a vicious cycle.

Notwithstanding these limitations, this research framework offers new perspectives and strategies for addressing practical issues related to problematic short-form video consumption. Specifically, by examining how individual traits, social environments, and platform features interact, this framework provides a more complete picture of why problematic use occurs. This can lead to more effective intervention strategies. For instance, identifying key distal factors that indirectly influence usage patterns through proximal factors allows for targeted interventions at multiple levels; intervention can be designed to modify psychopathology, usage expectations, and in-use responses, thereby reducing the risk of problematic use. Furthermore, understanding the impact of social-environmental and platform factors provides a basis for developing community-based and policy-level interventions. Schools and families can implement programs that foster healthy social environments, thereby mitigating negative influences. For example, the media consumption habits of parents and their subsequent guidance concerning their children’s use of short videos exert a substantial influence on adolescents’ media interaction patterns (56, 60). By monitoring their own use patterns and fostering robust communication channels within the family, parents can more effectively mentor their children in navigating short-form video platforms, thereby cultivating a foundation for healthy media consumption habits. In parallel, schools or educational institutions can play a pivotal role by integrating programs that facilitate a deeper understanding among users about the mechanisms behind content recommendation algorithms. Educating users about the functionality and objectives of these algorithms can enhance their vigilance regarding their content consumption patterns and improve selective engagement with content, thereby diminishing passive acceptance of recommendations. Additionally, platform designers can also use these insights to create features that promote healthier usage patterns and reduce addictive behaviors. Specifically, platforms can implement strategies to periodically prompt users to reflect on their viewing habits and suggest breaks or alternative activities, assisting them in recognizing and modifying potential problematic behaviors. Collectively, these strategies can contribute to a multifaceted approach that addresses problematic short-form video consumption through individualized, community-based, and technological interventions, fostering healthier digital environments and promoting well-being.

We encourage researchers to consider the proposed multidimensional framework when designing research on problematic use of short video platforms. Given that the existing studies reviewed in this paper are not without limitations, future studies could also consider improvements in the following areas:

First, adopting a longitudinal design. Existing research on problematic use of short video platforms predominantly employs cross-sectional designs, with only a few studies using repeated measures (40, 53). When employing a cross-sectional design, researchers are unable to determine whether the correlation between risk factors and problematic use reflects the predictive effect of the risk factors or the exacerbation of risks due to problematic use. To distinguish these possibilities, future research needs to adopt longitudinal designs and may consider focusing on the following aspects: 1) the prediction of problematic use of short video platforms by risk factors and their pathways of influence; 2) the dynamic interactions between risk factors and short video platforms (problematic) use; 3) the negative consequences triggered by problematic use of short video platforms.

Second, including diverse age groups. Existing studies predominantly focus on adolescents and young adults, leaving a gap in understanding the effects on other age groups. Future research should include a broader demographic range to examine whether the proposed framework holds across different age groups and to identify age-specific risk and protective factors.

Third, broadening the examination of variables. Future research could incorporate a wider range of variables, particularly those that have been less examined, such as the macro social environment within environmental factors and certain use modes (e.g., active versus passive use) within individual factors. As short video platforms continue to evolve with new features and technologies, future research should stay abreast of these changes (e.g., advanced algorithmic recommendations, problematic use prevention strategies) and investigate their impact on user behavior. Including these variables can provide a more comprehensive understanding of the diverse influences on problematic use of short video platforms.

Fourth, improving measurement of short video platforms use. Existing studies measuring (problematic) use of short video platforms rely on questionnaire-based, self-report assessments. Meta-analytical research shows that self-report measurements of social media use are only moderately correlated with logged measures (e.g., using apps to record usage data), suggesting that the accuracy of self-report measures for social media use is insufficient (64). Future research should consider combining questionnaires with diary methods to prospectively assess participants’ use of short video platforms over a subsequent period or integrate usage data recorded on participants’ mobile devices and applications to confirm their usage patterns (64, 65).

Fifth, uncovering the neural mechanisms influencing problematic use. To date, only one study has examined the impact of short video platforms (specifically, personalization) on users at the neural mechanism level (24). This study compared brain activation patterns in individuals when watching personalized recommendation videos versus general videos. The results suggest that personalized recommendations on short video platforms may enhance individual video-watching behavior by promoting self-referential processing, enhancing internal attention, and reward learning, providing a new perspective for understanding the mechanisms through which the characteristics of short video platforms lead to problematic use. Future research is needed to delve deeper into the neural mechanisms that trigger and maintain problematic use behaviors, and their mediating and moderating roles between other risk factors and problematic use.

## Footnotes

1 source: https://www.omnicoreagency.com/tiktok-statistics/, accessed August 2024.
